# Iron Therapy and Oxidative Stress

**DOI:** 10.1155/MBD.1997.137

**Published:** 1997

**Authors:** P. Geisser

**Affiliations:** Vifor (International) Inc. Research Department Rechenstr. 37 P.O. Box, CH-9001 St. Gallen Switzerland


### IRON THERAPY AND OXIDATIVE STRESS

P. Geisser

Vifor (International) Inc., Research Department,
Rechenstr. 37, P.O. Box, CH-9001 St.Gallen, Swutzerland

### 1. Introduction

It has been recognized more than one hundred years ago that atmospheric oxygen, **the breath of life**, apart from being beneficial, can be toxic to all living organisms. Today we know that in both, the beneficial and the harmful effects, the same types of **reactive oxygen species (ROS)** are involved. It is fair to say that these species constitute some sort of chemical interface between life and death.

In this interface iron is a double-edged sword, as it may cause **iron induced oxidative stress.** In moderate quantities and leashed to protein or other iron binding species, it is an essential element in all cell metabolisms and growth, but toxic when unleashed. The unique abilities of iron to vary oxidation state, redox potential, and electronic spin configuration in response to different ligand environments, are important criteria enabling iron to play multifunctional roles as a protein cofactor. For example, iron-containing proteins play fundamental roles in respiration, oxygen delivery to tissues, DNA synthesis, and regulation of the citric acid cycle. Concurrent with the evolution of complex iron-containing proteins, nature needed to design an efficient and non toxic means of controlling iron absorption, transport, and storage. **This is because the same physical properties that allow iron to act as an efficient cofactor in controlled redox chemistry, also allow iron to act as a potent toxin when not shielded from oxidatively susceptible biomolecules.**

Toxicity's and pathologies, associated with the oxidation of DNA, proteins, lipids, and carbohydrates are subjects of intensive research. Collectively, the oxidation of these molecules has been termed „**oxidative stress**", which can be defined as the oxidation of cellular components thereby introducing harm to that cell or tissue.

The aim of this report is to define the role of iron in oxidative stress by reviewing potential iron species, mechanisms of action, and preclinical and clinical situations, where oxidative stress is involved in relationship to the iron status. It will be shown how ROS are produced and which of reactions they can initiate on the molecular level and also to what kind of effects (side effects) ROS can cause in biological systems. A special focus is given to DNA damage and carcinogenesis, lipid peroxidation in pregnancy and prematures, $Fe^{2+}$ - induced cytotoxicity and injuries to gastric cells, immunology, neurotoxicity, iron overload, pro-oxidant effects of ascorbic acid, and erythropoietin activity. Mostly situations where iron therapy is often indicated. Therefore also a critical evaluation of today's used iron therapy will be given.

### 2. Chemistry

By definition, **reactive oxygen species (ROS)** are the species that appear in the series of four one-electron-uptake steps when an oxygen molecule, $O_2$, is reduced to water, $H_2O$. In the laboratory experiment this can be achieved by electrochemical reduction of $O_2$ in the aqueous solution of an inert electrolyte.

*This lecture was presented at the European Cooperation in Scientific Research and Technology (COST) and the Chemistry of Metals in Medicine (COMM) meeting in Louvaine-la-Neuve, Belgium 22. - 25. January 1997.*

$$O_2 \xrightarrow{e^-} {}^\bullet O_2^- \xrightarrow[+2H^+]{e^-} H_2O_2 \xrightarrow[-OH^-]{e^-} {}^\bullet OH \xrightarrow[+H^+]{e^-} H_2O$$

**Figure 1**: *The series of ROS emerging from the stepwise reduction of $O_2$ to $H_2O$. The dots in the two species ${}^\bullet O_2^-$ and ${}^\bullet OH$ indicate that they are radicals, i.e. they contain an uneven total number of electrons. The symbol of an electron is $e^-$.*

Figure 2 shows how iron species can promote the formation of ROS by chemical reactions. This has been verified *in vitro* as well as *in vivo* model systems such as cell cultures.

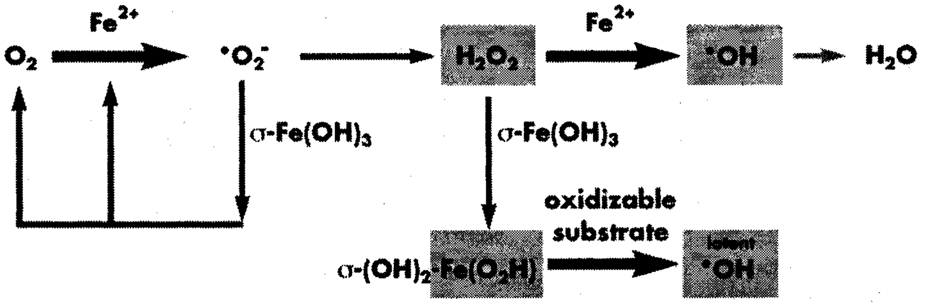

**The hydrated ferrous ion $Fe^{2+}$ and ferric hydroxide can interact in processes that lead to the formation of both free and latent hydroxyl radicals, •OH.**

**Figure 2**: *The hydrated ferrous ion $Fe^{2+}$ and ferric hydroxide can interact in processes that lead to the formation of both free and latent hydroxyl radicals •OH. The scheme is based on reaction (1), (2), and (3). The surface co-ordination of hydroperoxide $HO_2^-$ is the initial step in the well known disproportionation of $H_2O_2$ according to reaction (4), which is catalyzed by ferric hydroxides.*

$$Fe(OH)_3 + {}^\bullet O_2^- + 3H^+ \longrightarrow Fe^{2+} + O_2 + 3\,H_2O \quad (1)$$

$$Fe^{2+} + H_2O_2 + H^+ \longrightarrow Fe(III) + {}^\bullet OH + H_2O \quad (2)$$

$$Fe^{2+} + {}^\bullet OH + H^+ \longrightarrow Fe(III) + H_2O \quad (3)$$

$$H_2O_2 + H_2O_2 \longrightarrow 2\,H_2O + O_2 \quad (4)$$

Physiologically, in living organisms, the first reaction in Fig. 2 is rather irrelevant, as there are no fluids that contain an excess of ferrous ions, $Fe^{2+}$, in an oxygenated solution. **A deliberately caused exception to this situation results when ferrous salts are used in oral iron therapy.** The mucosal boundary is then exposed to gastric fluid with a fairly high concentration of ferrous ions, which immediately form superoxide radicals when they encounter oxygen molecules. This administration of iron(II) preparations therefore involves an artificial poisoning by reactive oxygen species, which is very similar to poisoning by radiation from radioisotopes (Günther 1990). However, many biochemists and pharmacologists believe that ferrous salt therapy is quite harmless.

### 3. Biochemistry

Localized within the mitochondria are the enzymes of the citric acid cycle, the electron transport arrangements, which delivers the electrons abstracted from intermediates of the citric cycle to oxygen, and the means by which the energy of this process is conserved by linking it to the formation of ATP. The first electron-acceptor of the **respiratory chain** is NAD which is reduced to $NADH_2$. The two H's, respectively the electron pair, reduce in the following steps flavoprotein (FP), Ubichinon (Coenzym Q), Cytochrom b + $c_1$, Cytochrom c and finally Cytochrom a + $a_3$. Cyto-chromoxidase and oxygen build $H_2O$

with two $H^{+}$. Thereby the energy is conserved in 3 moles ATP per mol $H_2O$. Each element of the respiratory chain has its own reduction potential, which allows a controlled reduction of $O_2$ to $H_2O$.
The concept of reduction potential is very useful in chemistry and biochemistry and is directly connected with the free energy of a redox reaction. Thereby the free energy, E, is **dependent on the stability of the oxidized and reduced species, and of the concentrations** of each reactant according to the following equation:

$$E = E^0 + \frac{R \bullet T}{n \bullet F} \ln \frac{C_{oxidzed}}{C_{reduced}}$$

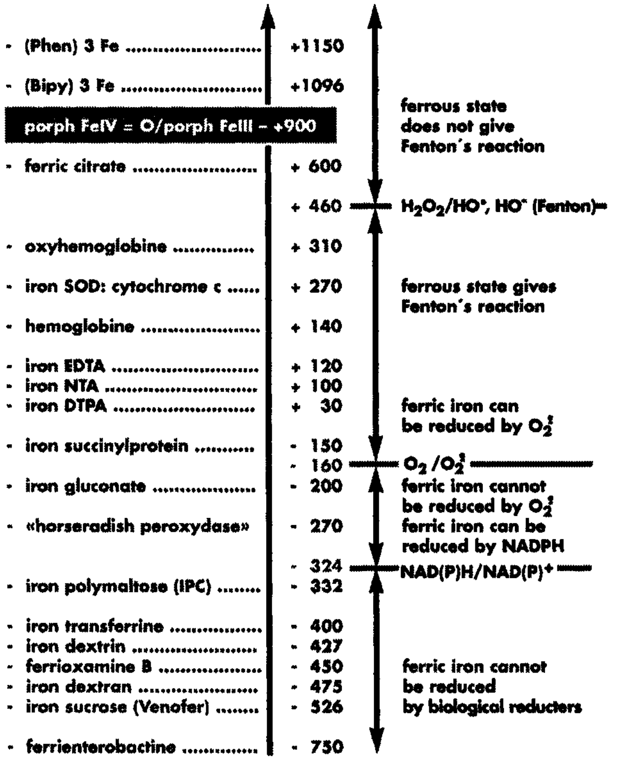

***Figure 3:*** *The reduction potentials of various iron(III)-compounds at pH 7.0 are listed, as well as the corresponding reactions, which can take place under physiological conditions. Fontecave et al. (1993), modified by Geisser (1997).*

### 4. Biological Observations

**Living organisms have developed defense systems which prevent or correct oxidative stress.**

***The primary defense system*** involves strict compartmentalization of molecules which are capable of catalyzing reactions with molecular oxygen.

***The second defense*** then, is provided by the dismutases, i.e. superoxide dismutases, which convert $^{\bullet}O_2^{-}$ to $O_2$ and $H_2O_2$, while the latter is transformed by catalase back to the innocuous molecules $O_2$, and $H_2O$.

However, there is also a **third defense system,** which is able to cope with $H_2O_2$ and with the most toxic hydroxyl radical $^{\bullet}OH$. This third system involves the intracellular removal of $H_2O_2$ by glutathione in conjunction with glutathione peroxidase, and the extracellular removal of $^{\bullet}O_2^-$ by ascorbate (vitamin C), or α-tocopherol (vitamin E).

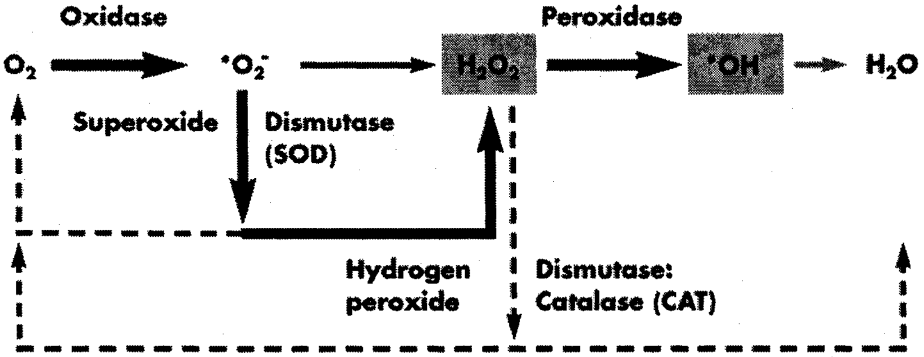

***Figure 4**: The superoxide ion is the source of the other reactive oxygen species $H_2O_2$ and $^{\bullet}OH$. The species, superoxide $^{\bullet}O_2^-$ and hydrogen peroxide $H_2O_2$ are removed by the enzymes superoxide dismutase (SOD) and catalase (CAT), respectively. The oxidases and peroxidases use oxygen for oxidative changes of organic substrates. Free $^{\bullet}O_2^-$ appear as by-products of oxidase activities. The most reactive species is the radical $^{\bullet}OH$. An important source of this radical is the Fenton reaction.*

The amount of ROS present in the tissue is determined by the rate of production (input) and removal (output) of ROS (Fig. 5; Moison et al. 1995). The **input** of $^{\bullet}O_2^-$ normally consists of production by mitochondrial and eicosanoid metabolism and by xanthine oxidase after ischemia/reperfusion (Berger et al. 1994). Activated neutrophils also produce $^{\bullet}O_2^-$, which dismutates to $H_2O_2$. Most of the $H_2O_2$ is converted to HOCl by myeloperoxidase (Gutteridge et al. 1989). The most reactive oxygen metabolite, $^{\bullet}OH$, is produced by decomposition of $H_2O_2$ in the presence of non-protein-bound transition metals such as Fe(II). However, non-protein-bound ferrous iron normally does not occur **(expect in cases of oral ferrous salt therapy)** since iron is maintained in the oxidized form by ceruloplasmin and rigorously bound to transferrin and ferritin (Gutterridge and Quinlan 1993). These proteins are referred to as primary antioxidants, since they prevent the production of ROS.

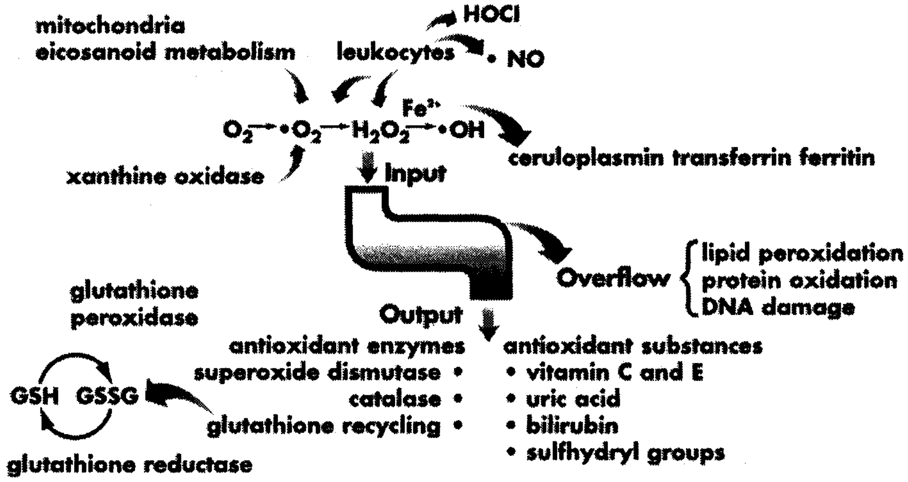

***Figure 5**: The input/output model of ROS shows factors which increase and defend oxidative stress and what results in an overflow situation. Moison et al. (1995).*

•NO is produced as an intercellular messenger by endothelium and neurons and plays important roles in vasoregulation and synaptic plasticity. Activated macrophages and neutrophils also produce •NO in similar rates as $^{\bullet}O_2$, and therefore the •NO can be converted into $ONOO^-$ (Beckmann et al. 1994).
The **output** of ROS depends on secondary antioxidants, consisting of antioxidant enzymes and antioxidant substances (Gutteridge et al. 1989). Superoxide dismutase converts $^{\bullet}O_2^-$ into $H_2O_2$, which in turn is reduced to water by catalase and glutathione peroxidase. The latter enzyme requires glutathione (GSH) as a substrate, which is oxidized to GSSG during $H_2O_2$ catabolism. Glutathione reductase reduces GSSG back to GSH, which in itself can scavenge $^{\bullet}O_2^-$ and •OH (Doelman et al. 1990). Other antioxidants such as vitamin C, vitamin E, uric acid, bilirubin, and protein sulfhydryl groups inactivate ROS (Halliwell et. al. 1990).
An increased production or decreased removal of ROS, i.e., an **imbalance between input and output**, results in oxidative damage of lipids, proteins and DNA. This leads to lipid peroxidation and membrane disruption, enzyme inactivation and sulfhydryl oxidation and DNA strand breakage. These processes result in celltoxicity, organ malfunction, and eventually in disease (Frank 1992).
**DNA strand breakage** was observed by Akasaka (1995) who found that $Fe^{2+}$ resulted in increased formation of 8-hydroxydeoxyguanosine in pZ189 DNA from Escherichia coli and increased supF mutations (up to 50%). This effect could be suppressed by catalase and SOD which is in agreement with the mentioned defense systems (see figure 4).

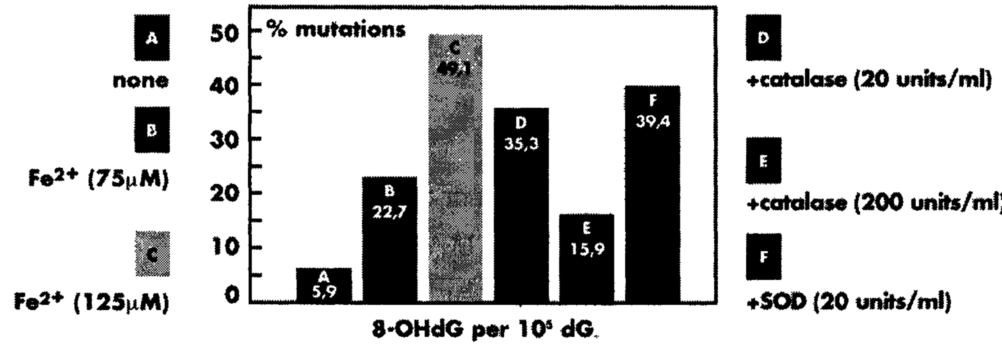

**50µg of plasmid pZ189 was incubated in 0,1 M potassium phosphate buffer (pH 7.4) with different concentrations of $Fe^{2+}$ at 37°C for 30 min.**

***Figure 6**: Induction of 8-Hydroxyguanosine formation by $Fe^{2+}$ and the effects of catalase and superoxide dismutase (SOD). Akasaka et al. (1995).*

Henle et al. (1996) and Luo et al. (1996) investigated the iron-mediated Fentons reaction under aerobic and anaerobic conditions to the deoxyguanosine and deoxycytidine families respectively. Due to the oxidative damage **20** degradation products of deoxyguanosine and **26** of deoxycytidine were found with the help of the HPLC technic. There was no difference in reactions between Fe(I) and Fe(II) species if the Fe(II) species could be reduced by NADH.
These results show that due to iron(II) oxidation •OH radicals cause serious damages to DNA constituents, and that the reduction potential is a good parameter for the characterization of iron(III) compounds.

### 5. Preclinical and Clinical

### 5.1 Lipid peroxidation

Marx et al. (1996) compiled a list of clinical conditions including more than 60 toxic states where reactive oxygen radicals are thought to be involved.
One of the most observed damages is **lipid peroxidation.** Fuhrman et al. (1994) showed that hydroxyl radical, but not superoxides or hydrogenperoxides, mediated the iron induced cellular lipid peroxidation. This means that SOD and catalase had no protective effect in case of J-774 macrophages, but also (and most important) that **$^{\bullet}OH$ radicals** and not $^{\bullet}O_2^-$ radicals or $H_2O_2$ are involved in lipid peroxidation (see figure 2).

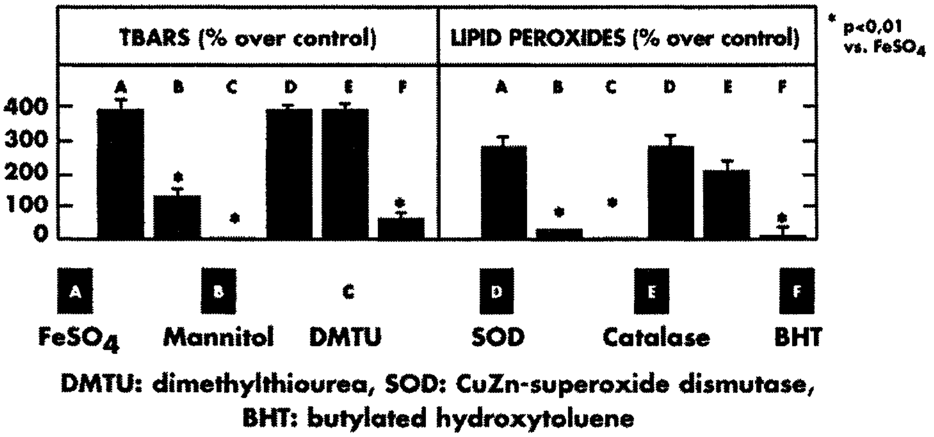

***Figure 7:** Involvement of free radicals in iron-induced cellular lipid peroxidation in J-774 macrophages . Fuhrmann et al. (1994).*

### 5.1.1 In Pregnancy

It has been reported that in **uncomplicated pregnancy** there is an increase of lipoperoxidation products in serum as gestation progresses (Hubel 1989). This finding correlates with changes in lipoperoxidative activity of the placenta (Cester 1994) and with an increase of total serum lipids. Controversial data is available on the antioxidant capacity in pregnancy. Stark et al. (1993) reported that lipid peroxidation is controlled by an adequate antioxidative response during normal pregnancy.

Wisdom et al. (1991) reported in PIH (Pregnancy induced hypertension) a decrease in plasma thiols and SOD and an increase in ceruloplasmin level, suggesting increased free radical activity. Whether this situation is a cause or an effect of oxidative stress in PIH has not yet been elucidated.

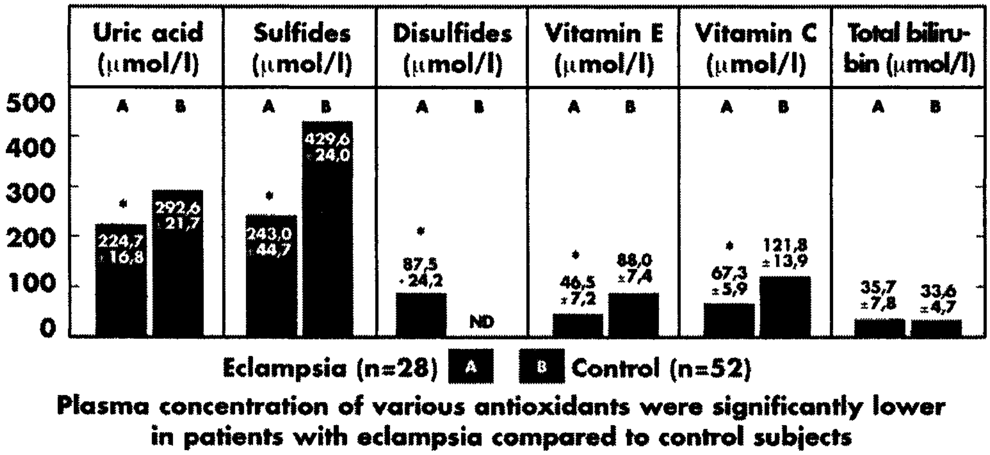

***Figure 8:** Plasma concentration of antioxidants in patients with eclampsia and the control group. Jendryczko et al. (1995).*

Jendryczko et al. (1995) found that the total radical trapping capacity of the antioxidants in plasma (TRAP) in patients with eclampsia, was lower than in the control group. Figure 8 shows a marked difference in sulfides and disulfides concentrations in comparison to the controls.
These findings suggest that oxygen free radical toxicity occurs in patients with eclampsia. The excess of free radicals may in turn be a factor that contributes to the complication of eclampsia. Davidge et al. (1992) reported similar results and showed that the antioxidant activity increases during gestation.

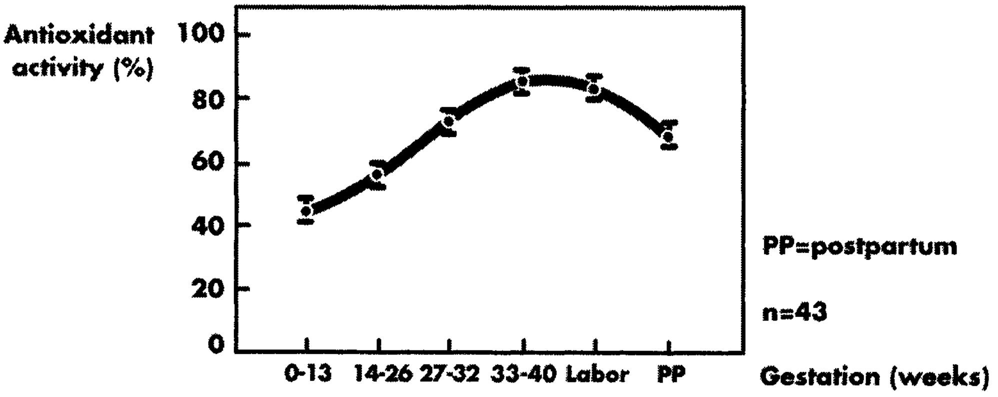

*Figure 9: Antioxidant activity in an uncomplicated pregnant population. Davidge et al. (1992).*

### 5.1.2 In Prematures and Children

In **premature infants** oxygen free radical mediated tissue injury is implicated as a major factor in the pathogenesis of observed long term complications. Inder et al. (1994) observed a relationship between lipid peroxidation, antioxidation activity, and outcome in cases of very low birthweight infants.

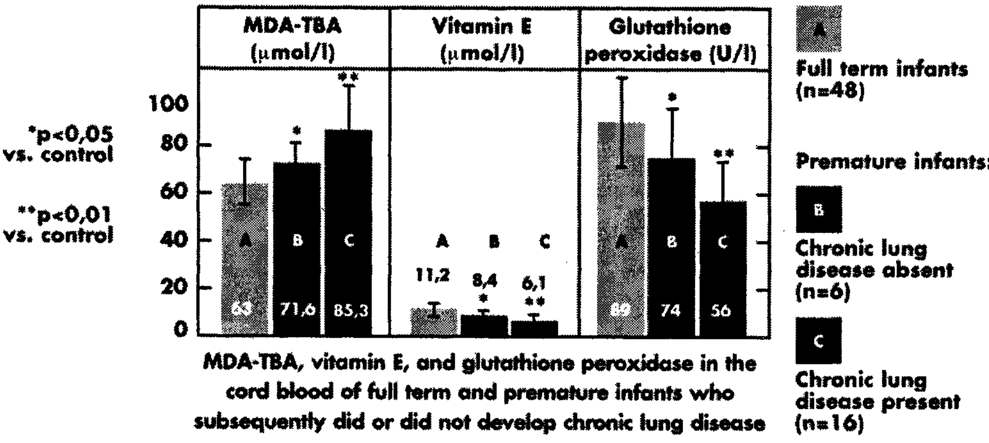

*Figure 10: Lipid peroxidation as a measure of oxygen free radical damage in very low birthweight infants. The results were obtained from the cord blood of full term and premature infants. Inder et al. (1994).*

Khaled et a. (1995) studied the influence of protein supply to malnourished children and found a decrease in oxidative stress parameters and an increase in RBP (retinol binding protein), prealbumin and albumin.

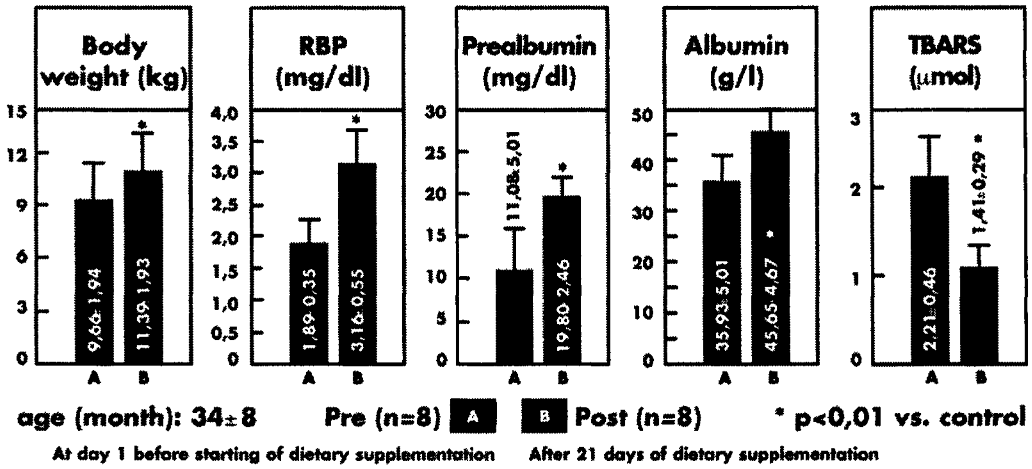

*Figure 11: Biochemical parameters of malnourished children before and after dietary supplementation. Khaled et al. (1995).*

In cases of **iron deficiency** in children, high levels of polyunsaturated fatty acids in erythrocytes membranes and an increased concentration of lipid peroxide (LPO) products (MDA and diene conjugates) in plasma and erythrocytes have been reported (Soboleva et al. 1994).

**Exactly in these situations with increased oxidative stress iron therapy is often required, but only the use of iron preparations without a further increase of oxidative stress will be adequate.**

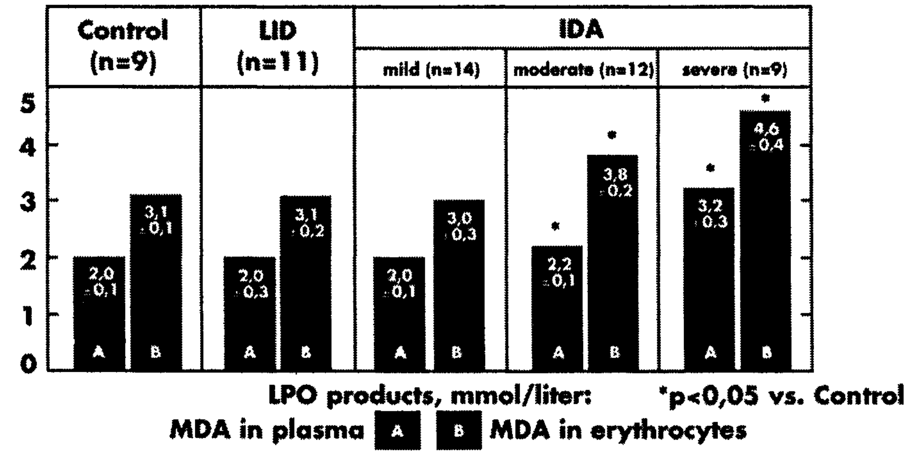

*Figure 12: Content of LPO products in plasma and erythrocytes in iron deficient in children (LID = latent iron deficiency, IDA = iron deficiency anemia). Soboleva et al. (1994).*

### 5.2 Toxicity

**The toxicity** of iron is explained by its ability to catalyze the formation of oxyradicals with resulting lipid peroxidation. Because of the high concentration of polyunsaturated fatty acids in their membranes, mitochondria appear particularly susceptible to peroxidation (Danielson et al. 1996, Geisser et al., 1992, Zimmermann 1988).

Hiraishi et al. (1993) found that **only $Fe^{2+}$**, e.g. produced by reduction of cellular $Fe^{3+}$, appears to be a prerequisite for the mediation of **damage in gastric epithelium.** This reduction is independent of extracellular $^{\bullet}O_2^-$ or cellular xanthine oxidase-derived $^{\bullet}O_2^-$. Further, the reaction of $H_2O_2$ with $Fe^{2+}$, presumablyforms a more reactive peroxo complex, which mayyield $^{\bullet}OH$ or higher oxidation states of iron (Haliwell1982; Sutton et al. 1989), leads ultimatelyto celldamage.

These results strongly reflect thefindings of Schaub et al. (1984) and Juji (1976). They found **serious gastric ulcers** and erosions after the application of ferrous sulfate to rabbits at a dosage of 230 mg Fe/kg body weight. With iron polymaltose complex at the same dosage no visible changes could be observed.

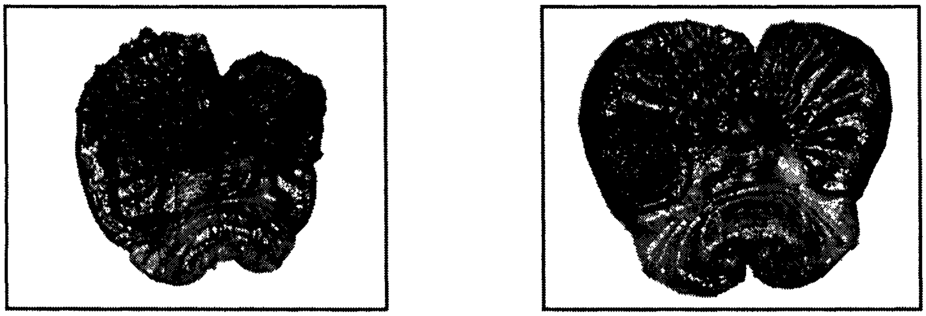

**After administration of Iron Polymaltose Complex (IPC Maltofer): No visible changes.**

**After administration of non-encapsulated Fe(II)-sulphate: Serious gastric ulcers and erosion of further areas.**

***Figure 13:*** *Stomachs of animals after administration of $FeSO_4$ and IPC (iron polymaltose complex). Schaub et al. (1984).*

Kadiska et al. (1995) investigated theformation of hydroxyl radicals by electron spin resonance (ESR) spectroscopy in rats with chronic dietary iron loading, using ferric citrate. The results show that ferric citrate leads to **radical generation in the bile**. This is in correlation with the redox potential of ferric citrate (+ 600 mV) (figure 3).

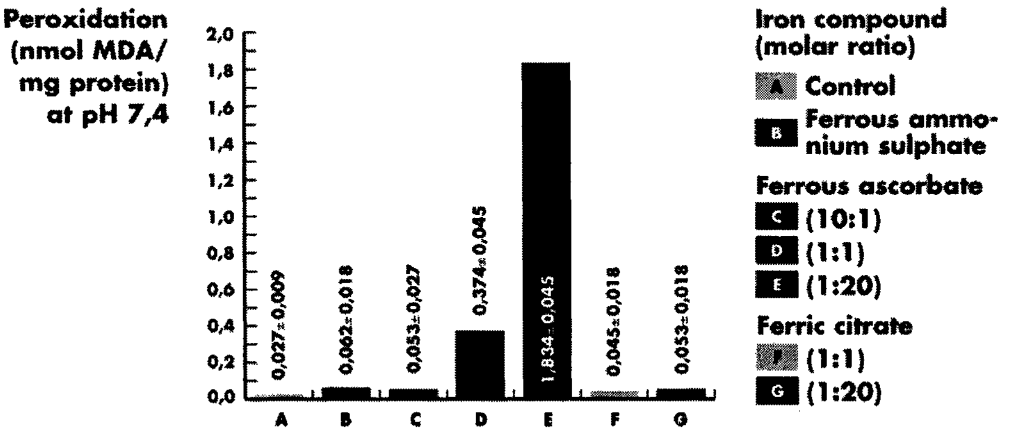

***Figure 14:*** *Peroxidation of rabbit smal intestinal microvillus membrane vesicles by various iron compounds. Fodor et al. (1988).*

Fukuda et al. (1996) found that Fe(II)-NTA induces **acute renal proximal tubular necrosis** in rats, a consequence of free radical-mediated oxidative tissue damage. A single intraperitoneal Fe(II)-NTA treatment (15 mg Fe/kg bodyweight) induced a production of MDA and the loss of sulfhydryl contents in the kidneys, resulting in a 30% reduction of glutathione S-transferases (GSTs).

Fodor et al. (1988) found that ferrous iron in a combination of 1:1 and 1:20 **with ascorbic acid** gives a high increase of MDA production at pH 7.4 in rabbits smal intestinal microvilles membrane vesicles.

Naito et al. (1995) developed a new gastric ulcer model and found that **ascorbic acid in combination with $Fe^{2+}$** produced significantly **more ulcers** than ascorbic acid or $Fe^{2+}$ alone.

The fact of severe intoxication of young children shows that toxicity of ferrous salts is a real clinical problem as published by the FDA 1994.

The number of young children who have accidentally swallowed iron has more than doubled since 1986. Since 1986, reports of more than 110 000 children under 6 who accidentally swallowed iron tablets were made to poison control centers. Many of the children were hospitalized, and at least 33 died.

### 5.3 ROS, Iron status/therapy and immunology

It is very interesting to note that ROS, **iron status,** and **iron therapy** influence **immunological paramaters.** The reactive oxygen species (ROS) have a central and important role in the activation of the transcription factor NF-κB, which is built up by two subunits p50 and p65 and held together by IκB. Different pathogenic and pro-inflammatoric stimuli, such as $H_2O_2$, different noxes (UV-irradiation, xenobiotica for example ferrous salts), T-cell mitogens, cytokines, and the infection with bacteria and viruses lead to a rapid activation of NF-κB. **All of them lead to a production of ROS.** As a result NF-κB is liberated, absorbed by the cell nucleus and bound to the DNA-sequences of the target genes. Such target genes are immune relevant genes, which code for cytokines, immune receptors, adhesion molecules and acute phase proteins. Viruses like HIV-1 use also NF-κB as a „host cell" for the replication of the virus genom (Blum 1996). This mechanism also explains the rapid increase of ferritin (active phase protein) after iron(II)-salt therapy. As show below, a ferritin increase does not always reflect an increase of depot iron (please see chapter 6).

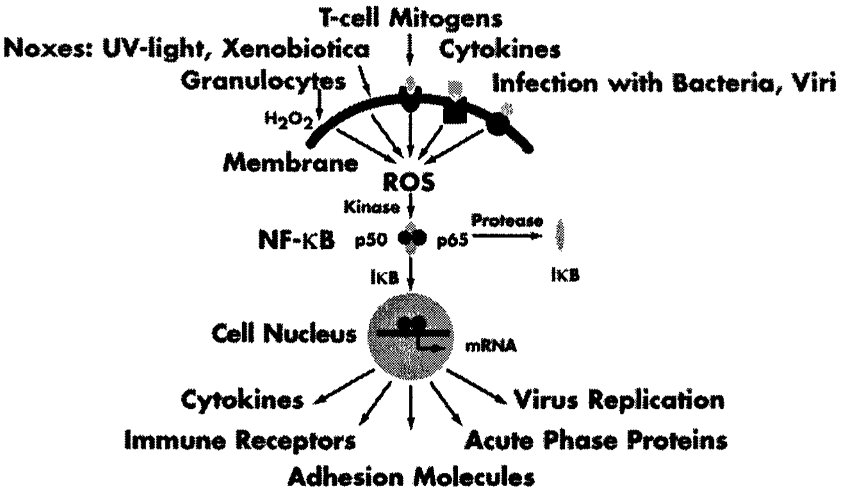

***Figure 15:*** *ROS plays a central role in the activation of NF-κB (Explanation see text) Blum (1996).*

Due to iron deficiency, the following immunological parameters will change (Santos et al. 1990)

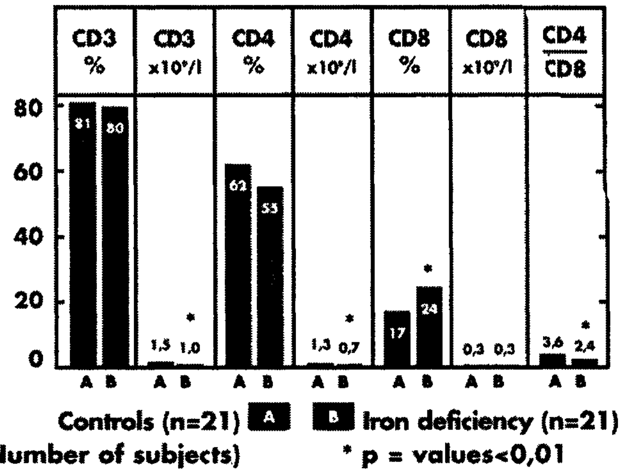

***Figure 16:** Partial immunological profile of patients with IDA (iron deficiency anemia). Santos et al. (1990).*

The effects of iron therapy on the different immunological parameters have to be carefully interpreted. This is because on the one hand, absolute and relative values can not always be compared, and on the other hand different iron preparations seem to have different effects. There is also an influence of concomitant diseases and the initial iron status before starting any therapy, e.g. the patients of Thibault et al. (1993) have not shown any effect on the iron parameters during therapy, so no effect on immunological parameters can be expected.

Zanni et al. (1989) found an increase of $CD8^+$ and a decrease of $CD4^+$ cells using iron(III)-protein succinate for iron deficiency therapy. That means that after normalization of the hematological parameters the immunological situation is still bad. This is because iron(III)-protein succinate can be reduced by physiological redox systems (cf. figure 3) to Fe(II)-species and as a result $^{\bullet}OH$ radicals can be produced, thus having a negative effect on CD4 cells.

Djeha et al. (1992) found that Fe(II)-NTA caused a decrease in the CD4/CD8 ratio, mainly due to the depression of the proportion of $CD4^+$ cells and a more modest increase in the proportion of $CD8^+$ cells. Since proliferation was also reduced under these conditions, the results may reflect a selective toxic or antiproliferative effect on the $CD4^+$ subset.

It is interesting to note that the iron compounds, having an inhibitory effect on the proliferative properties, also have a reduction potential which allows a reduction by $^{\bullet}O_2^-$ **or NAD(P) H, generating $Fe^{2+}$, and as a result $^{\bullet}OH$ radicals.**

### 5.4 Erythropoietin (EPO) activity and ROS

Said et al. (1995) showed that both Fenton and xanthine plus xanthine oxidase caused a significant reduction of EPO activity in mice. The treatment of EPO with $H_2O_2$ alone resulted in a relatively slight reduction of the activity.

The addition of chelating agents does not prevent the interaction with EPO as they only build stable complexes with Fe(III)- but not with Fe(II)-species.

As a result of these findings, iron preparations which induce the Fentons reaction should not be combined with EPO.

There is also some evidence that enzymatic antioxidant systems, represented by SOD and GPx, are decreased in erythrocytes of haemodialysis patients (Zima et al. 1996). In such situations iron(II)-salt therapy should be avoided.

Delmas-Beauvieux et al. (1995) found an increase of SOD, GPx and Cat in the erythrocytes in haemodialysed patients treated with EPO together with iron (in form of parenteral iron polymaltose complex) compared to single EPO or iron treatment. These results indicate the particular role of this therapeutic association in the induction of enzymatic defense via the increased rejuvenation of RBC. The findings are in agreement with the reduction potential of iron polymaltose complex, which shows that no iron(II) species, and as a result of this, no •OH radicals are formed (figure 3).

### 6. Effects of special Iron Complexes

Ciuffi et al. (1991) measured „in vivo" lipid peroxidation induction by different iron complexes in the brain cortex of rats. In the presence of 1 mM solution of ascorbic acid, **the generation of MDA (Malondialdehyd) was similar for iron polymaltose, iron sucrose and ferritin.**

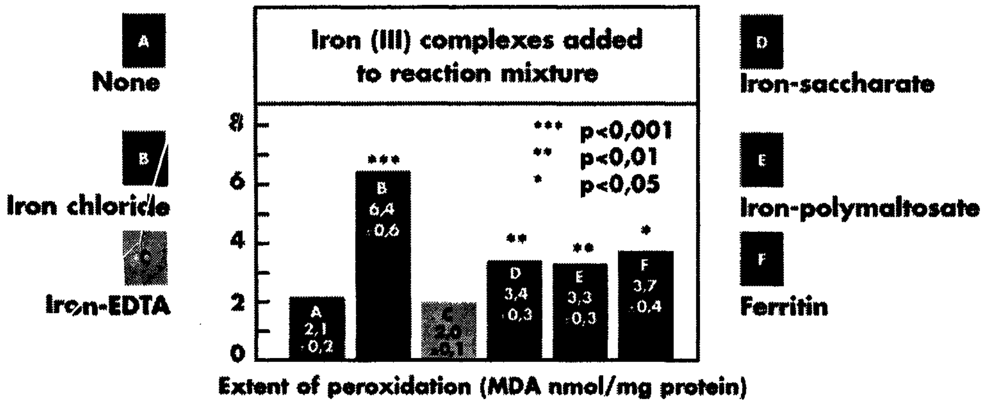

***Figure 17:*** *Iron(III)-complex-induced lipid peroxidation in rat brain cortex homogenate Ciuffi et al. (1991).*

This is in agreement with the measured redox potentials of these compounds (see figure 3). It was also shown that DL-α-tocopherol, methylprednisolone and D-penicilamine significantly decreased the iron induced lipid peroxidation, whereas desferrioxamine was not effective. This can be explained by the high stability of the investigated iron complexes (Geisser et al. 1992).

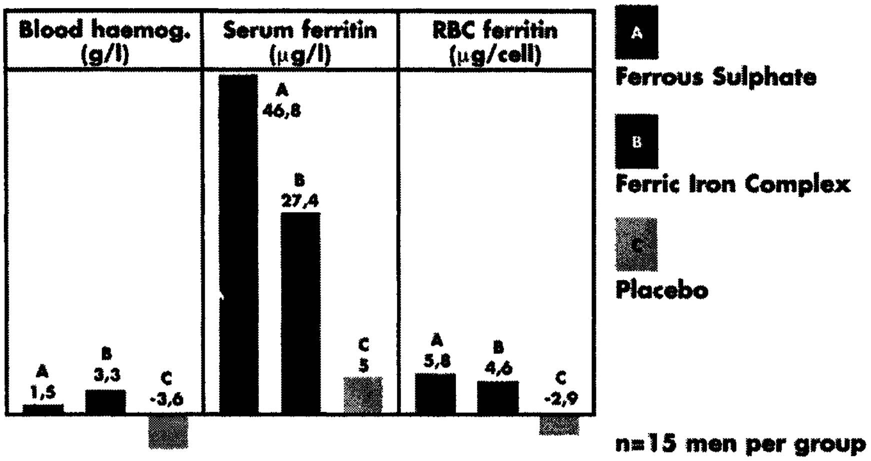

***Figure 18:*** *Changes in iron status in iron depleted male subjects after 6 months iron therapy with $FeSO_4$, iron polymaltose complex and placebo Salonen et al. (1996).*

Salonen et al. (1996) carried out a 6-month double blind trial evaluating the effects of iron supplementation in men with low iron stores with either ferrous sulfate or iron polymaltose complex. The susceptibility of very low and low density lipoproteins to oxidation increased in the ferrous sulfate group by 8.8% ($P < 0.05$) compared to the placebo group and by 12.8% ($p < 0.05$) compared to the iron polymaltose complex group. The amount of lipid peroxidation product increased due to ferrous sulfate by 13.8% ($p < 0.05$) compared to the iron polymaltose complex, with a more accentuated increase in hemoglobin levels in the iron polymaltose group (figure 18,19)
(Utilization studies using the twin isotope technic have shown that iron from iron polymaltose complex is equally utilized in humans and animals as from iron(II)-salts (Jacobs 1979, 1979, 1984)).

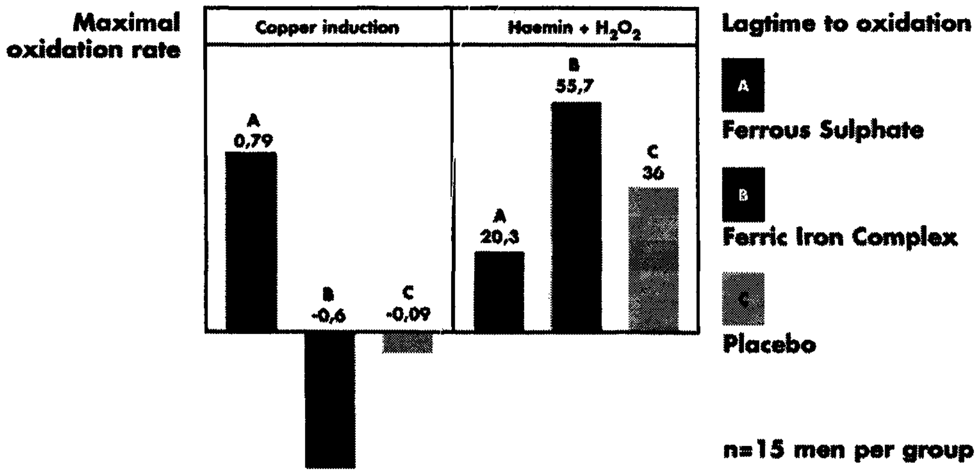

***Figure 19**: Changes in oxidation susceptibility in iron depleted male subjects after 6 months iron therapy with $FeSO_4$, iron polymaltose complex and placebo Salonen et al. (1996).*

These results show the role of rapidly absorbed and distributed ionic iron in oxidation susceptibility of lipoproteins in humans, and are in correlation with the redox potential of iron polymaltose as well as with the fact that $Fe^{2+}$ induces oxidative stress. The findings have important implications regarding the treatment of iron deficiency anemia. **They indicate that a rapid correction of iron stores by supplementation with ferrous sulfate, at least in combination with ascorbate, may have undesirable health effects and suggest that if iron supplementation is necessary, non-ionic iron polymaltose products are preferable to ferrous sulfate.**
It has to be mentioned that a ferritin increase does not always reflect an increase in depot iron. Cairo et al. (1995) have presented data which suggest that, under conditions of oxidative stress, liver ferritin can act either as a pro- or an anti-oxidant in a time-dependent manner. In fact its early degradation contributes to the expansion of the intracellular free iron pool that, later on, activates multiple molecular mechanisms to reconstitute ferritin content, thus limiting the pro-oxidant challenge of iron. It is obvious that iron therapy does increase the free iron pool. **In case of iron therapy with iron(II)-salts or with hem iron, not only is the free iron pool increased, but also the oxidative stress by the reaction of $Fe^{2+}$ with oxygen. As a result, a ferritin increase can be observed without a correlation with the amount of depot iron (see figure 44 Fig 44).**

### 7. Conclusion

Physicochemical data show that molecular oxygen develops a high reactivity especially in combination with trace elements for example Fe, Mn etc. by catalization of different biological oxidation processes. Thereby ROS (Reactive oxygen species) are produced, which can react with all kind of molecules yielding in the formation of denaturation products which can be toxic. It is shown that an input/output model is suitable to explain how much ROS are produced and detoxified in a so called steady state situation, and what kind of reactions will occur in case of overflow. The concept of reduction potentials for all types of redox processes involved leads to a clear and helpful instrument for the explanation and forecasting of such reactions.

Clinical data show that the so-called oxygen toxicity is unlikely to cause a number of pathological phenomena, unless the human (or animal) organism is burdened with excess **unphysiological** iron. Except for cases of genetic deficiency, oxygen toxicity appears to be man-made.

The most frequent observed damage is lipid peroxidation, which for example is found in cell membranes, fibroblast and macrophages, in pregnancy and prematures, in malnourished children and in children with IDA (iron deficiency anemia). It is a well documented fact that ferrous salts and ferric complexes with a reduction potential higher than that of NAD(P)H/NAD(P) lead to a great variety of undesirable effects in living organism: for example inference with ROS steady state and immunological parameters, toxic manifestations on kidney and bile, neurotoxic/neurodegenerative diseases, and DNA damages.

Undoubtfully there is a coincidence of status related oxidative stress for example in pregnancy, prematurity, IDA and the need of high iron supply. That means that the oxidative stress situation can be worsened by iron therapy, especially when the reduction potential is not considered. Iron overload, especially acute iron overload, increases oxidative stress parameters too. It is also a fact that ascorbic acid can develop a pro-oxidative effect in combination with iron. This leads to the recommendation that therapeutically used iron preparations should not be combined with ascorbic acid.

There is evidence that iron complexes of type I and II (for classification see Geisser et al. 1992), for example iron dextran, iron dextrin (iron polymaltose) and iron sucrose, do not provoke an increase of ROS production in living organisms. These complexes have very similar properties to ferritin and are therefore non-toxic.

| Type | Characteristic | Toxicity* | | Reduction potential | Preparations |
|---|---|---|---|---|---|
| | | Oral | Intravenous | | |
| Type I | robust and strong | > 2500 | >2500 | < -324 mV | Iron Dextran BP/USP<br>Iron Dextrin injectable |
| Type II | half robust and medium strong | > 2500 | > 200 | < -324 mV | Iron sucrose complex<br>Iron polymaltose complex |
| Type III | labile and weak | 429-1000 | 13-16.5 | > -324 mV | Iron(III)-gluconate<br>Iron(III)-citrate<br>Iron(III)-sorbitol |

* $LD_{50}$ white mice in mg Fe/kg bodyweight

Nevertheless for iron supplementation the most widely used form is ferrous sulfate as mentioned by Hider (1996) on the fifth international conference on disorders of iron metabolism. Although the bioavailability of Fe(II) (ferrous) salts is generally acceptable, many patients suffer side effects, resulting in poor compliance. Metal ions, including Fe(II), tend to induce an emetic effect which can be ameliorated by chelation. In most cases however, chelation reduces the absorption of the metal from the gut. An additional potential hazard with relatively high doses of Fe(II) salts is that they generate damaging hydroxyl radicals in the presence of vitamin C and oxygen. Unfortunately, the bioavailability of **Fe(III) salts** is generally much lower than that of the corresponding Fe(II) compounds, and consequently **ferric salts** are not widely used for supplementation purposes. Recently, however, two new **Fe(III) preparations** have been developed that possess acceptable bioavailabilities, namely **iron polymaltose** and **iron maltol**. These preparations may offer real advantages over ferrous sulfate, especially for those patients, who experience unpleasant side effects with iron(II) preparations (Hider 1996).

To avoid an increase of oxidative stress during iron therapy the following recommendations should be followed:

**No use of iron(II)-salts for therapy of iron deficiency and iron supplementation.**
**No use of ascorbic acid in combination with iron(II)-salts.**
**No use of iron(III)-compounds and complexes with reduction potentials higher than -324 mV at pH 7.**
**Use of only iron(III)-complexes with reduction potentials lower than -324 mV at pH 7 and with a similar substructure as ferritin.**

**Recommended compounds:**

**iron dextran**
**iron dextrin (iron polymaltose)**
**iron sucrose (saccharated iron oxide)**

### 8. Literature

1. Akasaka S, Yamamoto K. Biochemical and Biophysical Research Communications 1995;**213(1)**:74-80.
2. Beckmann JS, Chen J, Ischiropoulos H, Crow JP. Methods Enzymol. 1994;**233**:229-240.
3. Berger H M, Mumby S, Gutteridge JMC. Free Rad. Res. 1994;**22(6)**:555-559.
4. Berger HM, Moison RMW, van Zoeren-Grobben D. JR Col. Physicians Lond. 1994;**28**:24-33.
5. Berger HM, Mumby S, Gutteridge JMC. Free Rad. Res. Comms 1995;**22(6)**:555-559.
6. Blum HE. Dtsch. med. Wschr. 1996;**121**:1301-1302.
7. Cairo G, Tacchini L, Pogliaghi G, Anzon E, Tomasi A, Bernelli-Zazzera A. The Journal of Biology Chemistry, 1995;**270(2)**:700-703.
8. Cester N, Staffolani F, Rabini RA, Magnaelli R, Salvolini E, Galassi R, Mazzanti L, Romanini C.. Molecular and Cellular Biochemistry 1994;**131**:151-155.
9. Ciuffi M, Gentilini G. Franchi-Micheli S, Zilletti L. Neurochemical Research 1991;**16(1)**:43-49.
10. Danielson BG, Geisser P, Schneider W. *Iron therapy with special emphasis on intravenous administration 1996,* ***ISBN 3-85819-223-6.***
11. Davidge St, Hubel CA, Brayden RD, Capeless EC, McLaughlin MK. Obstetrics and Gynecology 1992;**79(6)**:897-901.
12. Delmas-Beauvieux M, Combe Ch, Peuchant E, Carbonneau MA, Dubourg L, de Précigout V, Aparicio M, Clerc M. Nephron 1995;**69**:404-410.
13. Djeha A, Brock JH. British Journal of Haematology 1992;**80**:235-241.
14. Doelmann CJA, Bast A. Free Rad Biol. Med. 1990;**9**:381-400.
15. FDA Medical Bulltin, February 1995/3.
16. Fodor I, Marx JJM. Biochimica et Biophysica Acta 1988;**961**:96-102.
17. Fontecave M, Pierre JL. Soc. Chim. Fr. 1993;**130**:77-85.
18. Fuhrmann B, Oiknine J, Aviram M. Atherosclerosis 1994;**111**:65-78
19. Fukuda A, Osawa T, Oda H, Toyokuni S, Satoh K, Uchida K. Archives of Biochemistry and Biophysics 1996;**329**:39-46.
20. Geisser P, Baer M, Schaub E. Arneim.-Forsch./Drug Res. 1992;**42(12)**:1439-1452.
21. Günther K. Naturwissenschaften 1990;**77**:412-420.
22. Gutteridge JMC, Halliwell B. Clarendon Press, Oxford 1989.
23. Gutteridge JMC, Rowley DA, Griffiths E, Halliwell B. Clin. Sci. 1985;**68**:463-467.

24. Halliwell B, Gutteridge JMC. Arch. Biochem. Biophys. 1990;**280**:1-8.
25. Halliwell B. Biochem. J. 1982;**205**:461-162.
26. Henle ES, Luo Y, Gassmann W, Linn St. The Journal of Biological Chemistry 1996:**271**:21177-21186.
27. Hider R ex, Bonkovsky HL et al. Hepatology 1996;**24**:718-729.
28. Hiraishi H, Ternao A, Razandi M, Sugimoto T, Harada T, Ivey KJ. Gastroenterology 1993;**104**:780-788.
29. Hubel CA, Roberts JM, Taylor RN, PhD, Musci TJ, Rogers GM, McLaughlin MK. Am. J. Obstet. Gynecol. 1989;**161**:1025-34.
30. Inder TE, Graham P, Sanderson K, Taylor BJ. Archives of Disease in Childhood 1994;**70**:F107-F111.
31. Jacobs P, Johnson G, Wood L. Journal of Medicine 1984;**15**:367-377.
32. Jacobs P, Wormald L. Journal of Medicine 1979;**10(4)**:279-185.
33. Jacobs P, Wormald L, Gregory M. South African Medical Journal 1979;**55**:1056-1072.
34. Jendryczko A, Tomala J. Zentralblatt Gynakol. 1995;**117**:126-129.
35. Kadiiska MB, Burkitt MJ, Xiang Q-H, Mason RP. The Journal of Clinical Investigation Inc. 1995; **96**:1653-1657.
36. Khaled MA, Kabir I, Mahalanabis D. Nutrition Research 1995;**15**:1099-1104.
37. Luo Y, Henle ES, Linn St. The journal of Biological Chemistry 1996;**271**:21167-21176.
38. Marx JJM, van Asbeck BS. Acta Haematol. 1996;**95**:49-62.
39. Moison RMW, Haasnoot AA, van Zoeren-Grobben D, Berger HM. *Pathogenesis and Detection of oxygen toxicity in the newborn; OxidativerStress in derKinderheilkunde*, Herausgeber H. Böhles Springer-Verlag 1995;**ISBN 3-540-59256-3.**
40. Naito Y, Yoshikawa T, Yoneta T, Yagi N, Matsuyama K, Arai M, Tanigawa T, Kondo MA. Digestion 1995;**56**:472-478.
41. Said AA, Morimoto K, Uchida E, Kawasaki N, Hibi K, Izaki Y, Hayakawa T. Free Rad. Res. 1995;**22**:229-238.
42. Salonen JT, Nyyssönen K, Porkkala-Sarataho E, Tuomainen TP, Geisser P, Salonen R. *Iron nutrition in health and disease*, edited by Leif Halberg and Nils-Georg Asp. 1996;John Libbey & Company Ltd.:pp293-301.
43. Santos PC, Falcao RP. Acta Haematol 1990;**84**:118-121.
44. Schaub E, Chovan K, Bürgin M, Renker H, Müller A. Hausmann Laboratories, Report 20.9.1984.
45. Soboleva MK, Sharapov, Grek OR. Bulletin of Experimental Biology and Medicine 1994;**117**:601-603.
46. Stark JM. British Journal of Obstetrices and Gynaecology 1993;**100**:105-109.
47. Thibault H, Galan P, Selz F, Preziosi P, Olivier C, Badoual J, Hercberg S. Eur. J. Pediatr. 1993;**152**:120-124.
48. Wisdom St.J, Wilson R, McKillop JH, Walker JJ. Am. J. Obest. Gynecol. 1991;**165**:1701-1704.
49. Zanni G. Ghini M, Mastrantoni M, Bonati ME. Current Therapeutic Research 1989;**45**:48-52.
50. Zima T, Stipek St, Crkovska J, Nemecek K, Platenik J, Bartova V, Tesar V. Blood Purif. 1996;**14**:257-261.
51. Zimmermann JJ. Critical Care Clinics 1988;**4**:645-660.

